# From tokenism to empowerment: progressing patient and public involvement in healthcare improvement

**DOI:** 10.1136/bmjqs-2015-004839

**Published:** 2016-03-18

**Authors:** Josephine Ocloo, Rachel Matthews

**Affiliations:** 1 Department of Surgery and Cancer, Faculty of Medicine, Centre for Patient Safety and Service Quality, Imperial College London, London, UK; 2 National Institute for Health Research (NIHR) Collaboration for Leadership and Applied Health Research and Care (CLAHRC) for Northwest London, Imperial College London, London, UK

**Keywords:** Healthcare quality improvement, Health policy, Patient-centred care

## Abstract

**Background:**

There have been repeated calls to better involve patients and the public and to place them at the centre of healthcare. Serious clinical and service failings in the UK and internationally increase the urgency and importance of addressing this problem. Despite this supportive policy context, progress to achieve greater involvement is patchy and slow and often concentrated at the lowest levels of involvement.

**Methods:**

A selective narrative literature search was guided by the authors’ broad expertise, covering a range of disciplines across health and social care, policy and research. Published systematic literature reviews were used to identify relevant authors and publications. Google and hand searches of journal articles and reference lists and reports augmented identification of recent evidence.

**Results:**

Patients and the wider public can be involved at most stages of healthcare, and this can have a number of benefits. Uncertainty persists about why and how to do involvement well and evaluate its impact, how to involve and support a diversity of individuals, and in ways that allow them to work in partnership to genuinely influence decision-making. This exposes patient and public involvement (PPI) to criticisms of exclusivity and tokenism.

**Conclusions:**

Current models of PPI are too narrow, and few organisations mention empowerment or address equality and diversity in their involvement strategies. These aspects of involvement should receive greater attention, as well as the adoption of models and frameworks that enable power and decision-making to be shared more equitably with patients and the public in designing, planning and co-producing healthcare.

## Introduction

Repeated calls have been made to engage and involve patients and the public and to place them at the centre of healthcare. Serious clinical and service failings in the UK^1^ ^2^ and internationally^3–5^ increase the urgency and importance of addressing this problem.^6–8^ Developing stronger patient and public involvement (PPI) in the organisation and delivery of healthcare is now central to health reform across Western economies.^9–11^ This recognition reflects evidence that patients and the wider public can be involved and make a difference at most stages of healthcare and in service planning and delivery.^12^ This, however, does not mean that all patients choose to be involved or indeed should have to be responsible for monitoring care, or indeed may not even be the most reliable way to do this, given their vulnerable condition.^13^ Despite this supportive policy context, progress to achieve greater involvement is patchy and slow and often concentrated at the lowest levels of involvement. By this we mean that consultation is more often the norm, than collaboration.^10^ ^14–16^ Some healthcare professionals and organisations have not embraced the idea of partnership with patients and even feel threatened by the notion of active involvement.^10^ ^17^ Though individuals, teams and organisations may be interested and deeply committed to involving patients and family members, they may lack clarity about what the issues are, who to involve and the goals of involvement.^18^ ^19^

## Methods

This article drew upon a selective narrative review^20^ of various sources of information and evidence connected to PPI. This was not meant to be a systematic review. We searched for literature up to March 2016 and omitted any literature published before 1969. The search was guided by the authors’ broad expertise and experience covering a range of disciplines from social work, health and social care, policy and research, clinical care and quality improvement. We used recent published systematic literature reviews to identify relevant authors and publications. Google and hand searches of journal articles and reference lists and reports augmented identification of recent evidence. Expert advice was sought from some cited authors. We selected literature that provided an overview of a range of arguments and methods about the benefits and difficulties with involvement and discussed conclusions. Personal experience in writing peer-reviewed publications in this field informed the analysis and synthesis of the overview.

## Results

A growing body of evidence suggests that patients can be involved and contribute to healthcare in various ways: from helping to reach an accurate diagnosis, choosing an appropriate treatment, management strategy or safe provider, ensuring treatment is properly adhered to and monitored and identifying adverse events and side effects and acting upon them.^12^ ^21^ Involving patients, their families and the public can also have a number of benefits: improving patient choice, self-care and shared decision-making (SDM) contributing to research partnerships and changes to service delivery and patient outcomes.^19^ ^22^ ^23^ The involvement process has also been seen as an important way in healthcare systems of enhancing democratic principles and accountability.^24^ ^25^ However, PPI often appears to be trapped in a vicious cycle. Uncertainty exists about why and how to do involvement well and how to involve and support a diversity of patients and the public, rather than a few selected individuals. The reality of implementation is complex and yields suboptimal evidence of impact.^22^ ^26^ This fuels the cycle of predictable and disappointing results and exposes PPI to criticisms of exclusivity and tokenism.^16^ ^27–29^ This article presents our reflections on these issues and explains why we think changing the balance of power, promoting empowerment, diversity and equality, and strengthening evaluation of outcomes and impact are the neglected aspects of involvement that, if given due attention, can offer a way to break the cycle.

### The purpose and value of involvement

Different words, theories and approaches have emerged from disparate social movements, policies and practices to describe the involvement process,^30^ for example, consultation, engagement, participation, partnership or co-production. These have sometimes been used to imply a greater or lesser level of involvement, power or influence in decision-making processes within an organisation. However, this language does not always reflect the underlying ethos of these involvement activities.^31^ In the absence of consensus on terminology,^30^ we define involvement as an activity that is done ‘with’ or ‘by’ patients or members of the public rather than ‘to’, ‘about’ or ‘for’ them.^32^ This definition sees the involvement process as a partnership between patients, the public and health professionals. This is important given major power differentials exist between those involved in a lay capacity and paid healthcare professionals.

**Figure 1:**
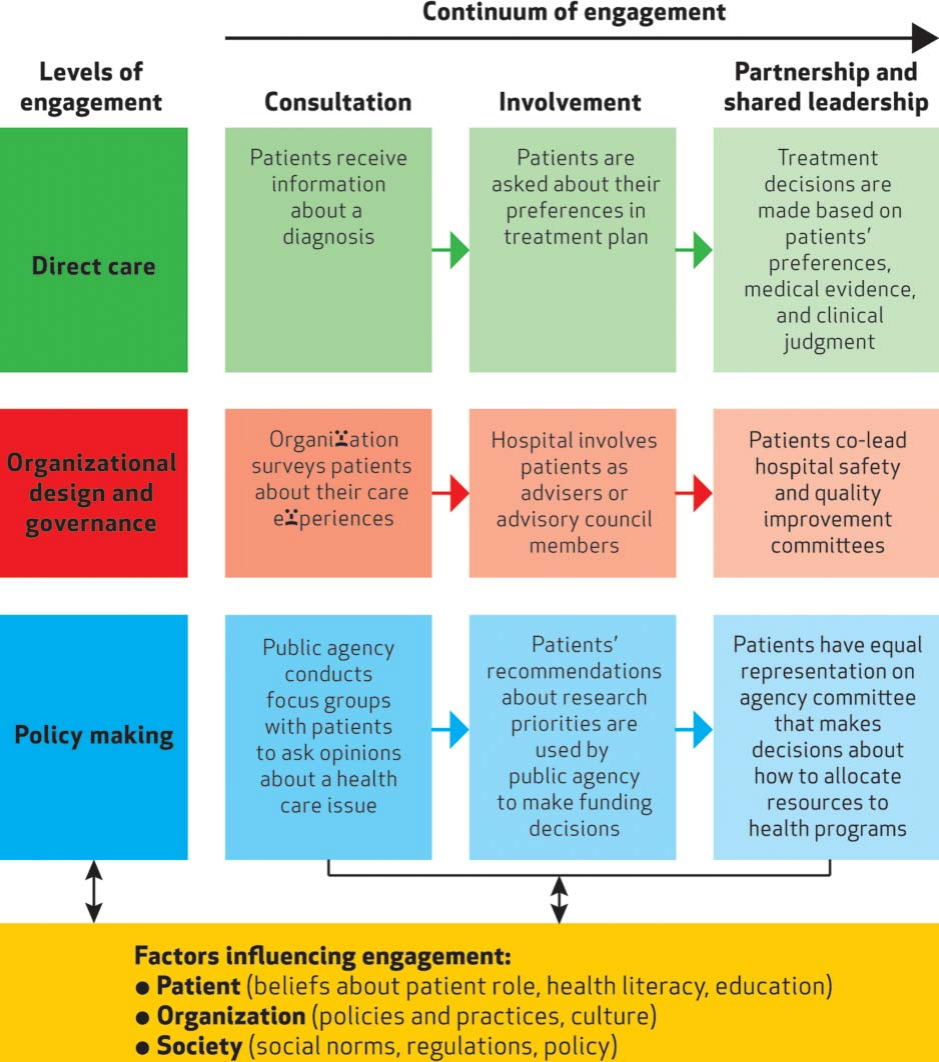
A multidimensional framework for patient and family engagement in health and healthcare.^33^ This figure builds upon Arnstein's^34^ widely quoted ‘ladder of citizen participation’. This described “a continuum of public participation in governance ranging from limited participation, or degrees of tokenism, to a state of collaborative partnership in which citizens share leadership or control decisions”.^33^ Reproduced with permission of Project HOPE/*Health Affairs* from Carman *et al.*^33^

At its core, the purpose of any involvement activity should be to improve the health and the experience of services for patients, their relatives, carers and users of health and social care services as well as the wider public.^31^ Figure 1^33^ provides a typical organising framework for involvement (the term engagement is used in this framework) that shows involvement can take place at multiple levels.

It can range along a continuum, from consultation to partnership and shared leadership. At the lower end, patients are involved but have limited power or decision-making authority. At the higher end, involvement is characterised by shared power and responsibility, with patients as active partners in defining agendas and making decisions. PPI can also occur at the level of individual health behaviour or direct care, or can occur at the collective level in organisational design and governance and in policymaking. We argue other areas can include commissioning, monitoring, evaluation and research. Multiple factors affect the willingness and ability of patients to engage at these different levels, including patient beliefs about their role, health literacy, education, organisational policies and practices and culture, society and social norms, regulation and policy. We believe that issues to do with inequality, discrimination and social exclusion also play a strong role in preventing many individuals and groups, participating in the involvement process as indicated in table 1.

**Table 1:** Summary of barriers and enablers for involvement^27^

Key exclusions	Key barriers	Overcoming barriers
*Equality and discrimination*: barriers on the basis of gender, ethnicity, culture, belief, sexuality, age, disability and class.	*Devaluing people*: not valuing or listening to what people say.	*Access*: ensuring all participants have effective ways into organisations and decision-making structures to have a real say in them.
*Where people live*: Homeless. In residential services. In prison and the penal system. Travellers/gypsies.	*Tokenism*: asking for involvement but not taking it seriously or enabling it to be effective.	*Support*: building confidence/skills, offering practical help/opportunities to get together to support people's empowerment and capacity.*Use of advocacy*: important for people who are disempowered and isolated.
*Communication issues*: Deaf people. Blind/visually impaired people. People who do not communicate verbally. People for whom English is not their first language. *Unwanted voices*: Some points of views/experiences are more welcome than others (particularly those who agree or are less challenging of the system or services). People can also be excluded because they are seen as too expensive/difficult to include such as those with dementia.	*Stigma*: stigmatising people for their identity or why they became involved or because they have had a poor experience of care^35^ and discouraging involvement on the basis of their identity. *Confidence and self-esteem*: making people feel they do not have much to contribute. *Inadequate information about involvement*: Lack of appropriate and accessible information about getting involved or about the involvement opportunities.^36^	*Different forms of involvement*: using innovative approaches that go beyond traditional methods: meetings, surveys, written and verbal skills. *Different methods include*: entertainment organised by lay participants, offering safe opportunities to explore ideas. Supportive activities, informal venues and encouraging networking. *Outreach and development work*: reaching out to those traditionally identified as ‘hard to reach’, going to them and community leaders, building trust, asking what works.*Meetings where used*: making them attractive, inclusive, enjoyable, with free refreshments that are culturally appropriate, safe, supportive environment, with access to key knowledge.
*Issues of poor health literacy*: this can be an important determinant of access to healthcare, impacting upon patients ability to book, cancel/attend appointments, respond to an adverse error in their care or medication or a deterioration in their care.^37^	*Gatekeepers/individuals who block the involvement process*: individuals who obstruct the involvement process by their attitudes or actions and stop people getting involved.	Tools to support patient empowerment^38^ *Motivational interviewing*: used by clinicians/non-clinicians, *personal budgets, expert patient* (self-management) *programme, patient decision aids* in shared decisions, *helping people prepare for consultations, access to health records.*
Achieving greater health literacy in the population is integral to improving the health of disadvantaged populations and to tackling health inequalities.^23^	*Financial barriers*: not paying participants for their involvement (which is a widely accepted principle) and speedily can deter people with limited resources or high costs because of the nature of their situation or impairment from being involved.	*Good practice regarding health literacy*^37^ Improving communication with all patients can include: *ascertaining what the patient knows* first to determine level of discussion. Speaking slowly, avoiding jargon, repeating points to improve comprehension, encourage and expect all patients to ask questions.*Check understanding and recall*. Ask patients to repeat back critical info (making clear this is about the health professionals’ ability to communicate clearly). *Communicate* in ways other than speech/printed material, eg, multimedia, translation services/materials.

### Understanding the role of power in involvement

Many authors capture the history and evolution of involvement and demonstrate that multiple influences and ideas have shaped this activity.^30^ ^39–41^ Global health social movements^42^ and disenfranchised groups, including black, disabled, mental health, lesbian and gay, and women's groups, can be seen as providing collective challenges to poor care and discriminatory or paternalistic services and medical policy and belief systems.^42^ These rights-based groups first emerged in healthcare and in other parts of the public sector in the UK as far back as the 1970s. Campaigns from patients who have been harmed during their care, and their relatives, are only the most recent manifestation of these challenges to paternalistic healthcare. These campaigns have been reinforced by wider arguments that also emerged in the late 1970s, emphasising the limitations of a biomedical model in promoting health and illness^43–45^ and calling for more shared models of treatment and decision-making.^46^ ^47^ Other concerns during this period highlighted a need to address poorer and unequal access to healthcare for some groups^48^ ^49^ as well as the role of the professions in healthcare iatrogenesis.^50^ ^51^ These arguments are still relevant today as we discuss later in the paper. In practice, what they illustrate are various examples where the balance of power apparently favours the organisation or system, rather than partnership working with patients and the public.^52^

With current involvement practice, power imbalances frequently manifest themselves in different ways, starting with who to involve. PPI often involves a narrow group of individuals, with the handpicking of just one or two ‘appropriate’ or ‘acquiescent’^29^ patient representatives to be involved in committees or projects. Patient representatives are less commonly drawn from black and minority ethnic groups,^27^ ^53^ ^54^ and are often middle class.^9^ ^29^ ^31^ Yet in the UK,^53^ ^55^ and “in most healthcare systems, it is acknowledged that black and minority ethnic (BME) populations have until now experienced poorer health and barriers to accessing certain services”.^56^ ^57^ Many other different groups are also excluded from involvement processes (table 1). These groups may have particular or even greater healthcare needs than the wider population,^58^ ^59^ yet their views are seldom heard or listened to.^16^ ^27^ ^28^ ^60^ In reality, the capacity to be successfully involved is significantly affected by education level, income, cognitive skills and cultural differences, which can affect patients’ health beliefs and ability to use health services.^12^ ^61^ The consequences of narrow PPI selection processes mean that those with most to gain are most excluded from healthcare decision-making. This restricts the pool of ideas for improvement and limits the opportunity to break cycles of suboptimal care and services.

At the organisational level, factors that can hinder PPI in service planning and decision-making include laypeople feeling unclear about their role and what is expected of them, a shortage of resources to support the process, concerns about representation, negative attitudes^27^ and resistance from healthcare staff and managers^12^ ^17^ ^29^ ^62^ (table 1).

In the UK, it has been suggested that the majority of involvement activity in healthcare has traditionally taken place at the level of feedback and information giving.^63^ Shared forms of decision-making, which have been found to have proven health benefits,^64^ are still not the norm.^65^ Internationally, there is evidence that shows that patient representatives are struggling to influence decisions and are largely expected to work within existing systems in improving quality and safety.^61^ Involvement at this level has been criticised as providing little opportunity to influence decision-making processes in any depth. This serves to maintain professional and system interests and power.^10^ ^34^ ^47^

Current models of PPI are therefore too often rooted in a mechanistic, controlled and professionally dominated approach, based upon a very practical and atheoretical way to getting someone's input. This narrow ‘managerialist or consumerist’ model^10^ has its roots in market research and ‘improving the product’, which has typically come to dominate approaches to PPI. It mainly draws upon data collection methods and consultation and the reporting of patient survey data at board meetings. This contrasts with a wider democratic rights and values-based approach, which emphasises the need for the direct involvement and empowerment of users of services in the decision-making process and broader democratisation at a community level. Such an approach goes beyond just a focus on individuals as the source of the problem and recognises the systemic nature of health inequities and how different groups can be excluded. Democratic models focus on the need for change to take place within social systems as well as within individuals and services.^66^

### Linking theory and evidence for empowerment and impact

Broader frameworks and methods of involvement should be used that offer better ways to share power with healthcare professionals. Central goals of involvement should focus on issues of inclusivity and representation, equalities, non-discrimination and empowerment. It has been recognised that different levels of participation are appropriate in different circumstances.^63^ But it is clearly important to think about which level is important and how it will influence decision-making. In current PPI practice, there appears to be a considerable disconnection with much of this thinking and how it can be used to achieve clarity of purpose in much of mainstream healthcare. This situation is worse in areas such as patient safety, where PPI is largely atheoretical.^61^

At the organisational and community level, models of co-production are increasingly being seen as a way of addressing power imbalances by designing and delivering public services in more democratic, equal and reciprocal relationships between professionals, people using services, their families and their neighbours.^67^ ^68^ There is no one ‘correct’ way of doing co-production, but there are six principles that help to underpin practice:

- assets: recognising people as assets;
- capabilities: building on people's existing strengths;
- mutuality: reciprocal relations with mutual responsibilities and expectations;
- networks: peer support and engaging a range of networks inside and outside services;
- blur roles: removing tightly defined boundaries between professionals and recipients to enable shared control and responsibility;
- catalysts: shift from delivering services to supporting things to happen.^67^ ^69^ ^70^

This more collaborative framework can also support methods to empower patients at the individual level. Evidence on four key ways to empower patients has been identified: (a) empowering individuals in their own care; (b) reviving the revolution in decision-making tools as part of a systematic drive in shared decision-making (SDM) (SDM offers a process where patients and clinicians can work together to select tests, treatments, access personal health records and health budgets, care planning and decision aids, management or support packages based on clinical evidence and the patient's informed preferences).^64^ ^65^ The latter is particularly important in preventing misdiagnosis and unwanted interventions.);^71^ (c) giving patients co-ownership of their records, not just access; and (d) encouraging patients to ask more questions and targeting a national campaign for people with long-term conditions and offering greater access to structured education on self-management. Tackling health literacy has also been found to be central to the empowerment of patients and reducing health inequalities.^23^ Table 1 provides examples of tools and ways to develop empowering practice with a diversity of groups at the individual and organisational level.

Empowering users and providers and supporting frontline staff to feel confident in sharing power and accepting user expertise will be crucial in developing these more shared and collaborative ways of working.^72^ This is important given that time, resources and funding^17^ and competing organisational priorities and a lack of training for clinical providers have been identified as key barriers in the implementation of PPI strategies.^73^

Finding effective ways to evaluate PPI processes are also important to ensure a wider range of expertise and experiences are included in PPI activities. There are a number of factors that make evaluation in this area challenging: the need for a shared understanding of PPI in practice and how it is conceptualised and measured, limited documentation of underpinning theory^19^ and the difficulty in isolating involvement from other factors that influence change.^18^ Within the democratic tradition, less emphasis has traditionally been placed on measuring PPI as involvement is seen as something that has intrinsic value in and of itself, over and above any attempt to measure it from an instrumental perspective. We suggest national involvement standards such as 4Pi^31^ (table 2) could provide a broader, inclusive framework by which to support good practice in the development of PPI approaches and interventions as well as to understand the effects of PPI. 4Pi draws on research evidence and user experience to identify the characteristics and attributes of meaningful involvement. This framework, in combination with a sound understanding of evaluation principles,^74^ could support the generation of better evidence.

**Table 2:** 4Pi national involvement standards: involvement for influence^31^

Component	Supportive attributes for inclusion and empowerment
Principles	Involvement is underpinned by values, eg, inclusivity and non-discrimination, respect and transparency and being open-minded to cultural differences.
Purpose	The purpose should be clearly articulated so that everybody understands the goal of involvement and has the opportunity to shape and influence the process.
Presence	Who to involve will be determined by the purpose. This means an inclusive approach that seeks to address inequalities.
Process	Involvement in direct care for example, will require different approaches to involvement than at the collective/organisational level. Consultation methods will deliver different results by comparison with co-design or co-production approaches.
Impact	Impact can be considered in different ways, eg, on individual conduct as well as on organisational culture, policy/planning, outcomes, outputs, diversity and equality of opportunity and the experience of service users.

## Conclusions

Our findings point to a need to re-evaluate methods and approaches for involving patients and the public in all aspects of healthcare and in healthcare improvement. Partnership working has long been the explicit stated goal of involvement. However, current involvement practices at a national and local level often involve a narrow group of individuals in involvement activities, with little consideration given to including a broader demographic of the population. Moving beyond this tokenistic, narrow and exclusive approach requires a critical appraisal of evidence and a debate about the focus and methods of involvement. Use of broader and more democratic models is important to address imbalances of power between patients, public and healthcare professionals and organisations. Evaluating these approaches, to understand the impact and effectiveness of chosen PPI methods, as well as how inclusive they are, is important. Developing greater partnership working will require key policy organisations and networks to take a lead in promoting this broader approach, disseminating good practice and evidence and building in requirements into funding streams.

Ultimately to deliver a broader and more effective approach to involvement, staff will need to be trained and supported within organisational contexts where partnership working with a diversity of patients and the public is clear, embedded and normal. Moving beyond tokenism to sharing power and decision-making more equitably will promote empowerment and help develop models of healthcare that are more co-designed and co-produced between all stakeholders, regardless of whether they are using or providing services.

## References

[1] Bristol Royal Infirmary Inquiry. The Report of the Public Inquiry into Children's Heart surgery at the Bristol Royal Infirmary 1984–1995. London: HMSO, 2001.

[2] Francis R Report of the Mid Staffordshire NHS Foundation Trust Public Inquiry. London: Stationery Office, 2013.

[3] Kohn LT, Corrigan JM, Donaldson MS To err is human: building a safer health system. Washington: National Academies Press, 1999. 25077248

[4] WHO. World Alliance for Patient Safety forward programme. Geneva: World Health Organization, 2008–2009.

[5] Walshe K, Shortell SM When things go wrong: how Health Care Organizations deal with major failures. Health Aff (Millwood) 2004;23:103–11. 10.1377/hlthaff.23.3.103 15160808

[6] WHO. European regional patients for patient safety workshop report. Geneva: World Health Organization, 2007.

[7] (APPG) APPG. Patient empowerment: for better quality, more sustainable health services globally. London: House of Commons, 2014.

[8] Edgman-Levitan S, Brady C, Howitt P Partnering with patients, families, and communities: a global imperative. Doha: World Innovation Summit for Health (WISH), 2013.

[9] Church J, Saunders D, Wanke M, et al Citizen participation in health decision-making: past experience and future prospects. Health Policy 2002;23:12–32. 12013713

[10] Tritter JQ Revolution or evolution: The challenges of conceptualizing patient and public involvement in a consumerist world. Health Expect 2009;12:275–87.. 10.1111/j.1369-7625.2009.00564.x 19754691 PMC5060496

[11] Wait S, Nolte E Public involvement policies in health: exploring their conceptual basis. Health Econ Policy Law 2006;1:149–62. 10.1017/S174413310500112X 18634687

[12] Coulter A, Ellins J Patient focused interventions: a review of the evidence. London: The Health Foundation, 2006.

[13] Lyons M Should patients have a role in patient safety? A safety engineering view. QSHC 2007;16:140–2. 10.1136/qshc.2006.018861 PMC2653153 17403763

[14] Parsons S, Winterbottom A, Cross P, et al The quality of patient engagement and involvement in primary care. London: The King's Fund, 2010.

[15] Healthcare Commission. Listening, learning, working together? London: Healthcare Commission, 2009.

[16] National Institute for Health Research. Going the Extra Mile: improving the Nation's health and wellbeing through public involvement in research. London: National Institute for Health Research, 2015.

[17] Brett J, Staniszewska S, Mockford C, et al A systematic review of the impact of patient and public involvement on service users, researchers and communities. Patient 2014;7:387–95.. 10.1007/s40271-014-0065-0 25034612

[18] Crawford MJ, Rutter D, Manley C, et al Systematic review of involving patients in the planning and development of health care. BMJ 2002;325:1263 10.1136/bmj.325.7375.1263 12458240 PMC136920

[19] Mockford C, Staniszewska S, Griffiths F, et al The impact of patient and public involvement on UK NHS health care: a systematic review. Int J Qual Health Care 2012;24:28–38. 10.1093/intqhc/mzr066 22109631

[20] Green B, Johnson C, Adams A Writing narrative literature reviews for peer-reviewed journals: secrets of the trade. J Sports Chiropr Rehabil 2001;15:5–19. 10.1016/S0899-3467(07)60142-6 PMC2647067 19674681

[21] Vincent CA, Coulter A Patient safety: what about the patient? Qual Saf Healthc 2002;11:76–80. 10.1136/qhc.11.1.76 PMC1743559 12078376

[22] Brett J, Staniszewska S, Mockford C, et al Mapping the impact of patient and public involvement on health and social care research: a systematic review. Health Expect 2012;17:637–50. 10.1111/j.1369-7625.2012.00795.x 22809132 PMC5060910

[23] Coulter A, Ellins J Effectiveness of strategies for informing, educating and involving patients. BMJ 2007;335:24–7. 10.1136/bmj.39246.581169.80 17615222 PMC1910640

[24] Irvine D The Doctors’ Tale. London: Routledge, 2004.

[25] Davies HTO, Shields AV Public trust and accountability for clinical performance: lessons from The National press reportage of the Bristol Hearing. J Eval Clin Pract 1999;5:335–42. 10.1046/j.1365-2753.1999.00200.x 10461585

[26] Staniszewska S, Adebajo A, Barber R, et al Developing the evidence base of patient and public involvement in health and social care research: the case for measuring impact. Int J Consum Stud 2011;35:628–32. 10.1111/j.1470-6431.2011.01020.x

[27] Beresford P Beyond the usual suspects. London: Shaping Our Lives, 2013.

[28] Trivedi P Black service ‘user involvement’—rhetoric or reality. In: Fernando S, Keating F, eds. Mental Health in a Multi-Ethnic Society. 2nd edn. London: Routledge, 2009:136–46.

[29] Martin GP Representativeness, legitimacy and power in public involvement in health-service management. Soc Sci Med 2008;67:1757–65. 10.1016/j.socscimed.2008.09.024 18922611

[30] Gibson A, Britten N, Lynch J Theoretical directions for an emancipatory concept of patient and public involvement. Health (London) 2012;16:531–47. 10.1177/1363459312438563 22535648

[31] Faulkner A, Yiannoullou S, Kalathil J, et al Involvement for Influence. 4PI National Involvement Standards. London: National survivor User Network (NSUN), 2015.

[32] INVOLVE. Briefing notes for researchers: involving the public in NHS, public health and social care research. Eastleigh: Hampshire INVOLVE, 2012.

[33] Carman KL, Dardess P, Maurer M, et al Patient and family engagement: a framework for understanding the elements and developing interventions and policies. Health Aff (Millwood) 2013;32:223–31. 10.1377/hlthaff.2012.1133 23381514

[34] Arnstein SR A ladder of citizen participation. J Am Plann Assoc 1969;35:216–24.

[35] Ocloo JE Harmed patients gaining voice: challenging dominant perspectives in the construction of medical harm and patient safety reforms. Soc Sci Med 2010;71:510–16. 10.1016/j.socscimed.2010.03.050 20554367

[36] Ocloo JE, Fulop NJ Developing a critical approach to patient and public involvement in patient safety in the NHS: learning lessons from other parts of the public sector? Health Expect 2012;15:424–32. 10.1111/j.1369-7625.2011.00695.x 21711471 PMC5060634

[37] Royal College of General Practitioners. Health Literacy. London: Royal College of General Practitioners, 2014.

[38] National Voices. Supporting shared decison-making. Secondary Supporting shared decison-making 11th September 2015 http://www.nationalvoices.org.uk/evidence

[39] Andersson E, Tritter J, Wilson R Healthy Democracy: the future of involvement in health and social care. London: Involve and NHS National Centre for Involvement, 2012.

[40] Coulter A Engaging patients in healthcare. Maidenhead: Open University Press, 2011.

[41] Barnes M, Cotterell P Critical perspectives on user involvement. Bristol: The Policy Press, 2012.

[42] Brown P, Zavestoski S Social movements in health: an introduction. Sociol Health Illn 2004;26:679–94. 10.1111/j.0141-9889.2004.00413.x 15383036

[43] Freidson E Professional dominance: the social structure of medical care. Chicago: Aldine Publishing Company, 1970.

[44] Oakley A ‘Wisewoman and medicine man: changes in the management of childbirth. In: Mitchell J, Oakley A, eds. The rights and wrongs of woman. Harmondsworth: Penguin, 1976:17–58.

[45] Rogers A, Pilgrim D Pulling down churches: accounting for the British mental health users’ movement. Sociol Health Illn 1991;13:129–48. 10.1111/1467-9566.ep11340759

[46] Charles C, Gafni A, Whelan T Decision-making in the physician patient encounter: revisiting the shared treatment decision-making model. Soc Sci Med 1999;49:651–61. 10.1016/S0277-9536(99)00145-8 10452420

[47] Rutter D, Manley C, Weaver T, et al Patients or partners? Case studies of user involvement in the planning and delivery of adult mental health services in London. Soc Sci Med 2004;58:1973–84. 10.1016/S0277-9536(03)00401-5 15020013

[48] Acheson D Independent Inquiry into inequalities in health report. London: The Stationery Office, 1998.

[49] Townsend P, Davidson N, Whitehead M Inequalities in health: the black report and the health divide. London: Penguin, 1988.

[50] Illich I Medical Nemesis. Lancet 1974;1:918–21. 10.1016/S0140-6736(74)90361-4 4133432

[51] Schon D The reflective practitioner. London: Temple Smith, 1983.

[52] Donaldson L The challenge of quality and patient safety. J R Soc Med 2008;101:338–41. 10.1258/jrsm.2008.nh8003 18591685 PMC2442132

[53] Stuart O User participation in health care services. London: Race Equality Foundation, 2008:1–7.

[54] Boote J, Wong R, Booth A ‘Talking the talk or walking the walk?’ A bibliometric review of the literature on public involvement in health research published between 1995 and 2009. Health Expect 2012;18:44–57. 10.1111/hex.12007 23033933 PMC5060762

[55] Fernando S Multicultural mental health services: projects for minority ethnic communities in England. Transcult Psychiatry 2005;42:420–36. 10.1177/1363461505055624 16268236

[56] Bécares L Which ethnic groups have the poorest health? Ethnic health inequalities 1991 to 2011. Manchester: University of Manchester, Joseph Rowntree Foundation, 2013.

[57] Szczepura A Access to health care for ethnic minority populations. Postgrad Med J 2005;81:141–7. 10.1136/pgmj.2004.026237 15749788 PMC1743229

[58] White S What does good care look like for a deafblind person?. London: Sense, 2014.

[59] Mencap. Death by indifference. London: Mencap, 2007.

[60] Beresford P, Branfield F Building solidarity, ensuring diversity: lessons from service users’ and disabled people's movement. In: Barnes M, Cottererell P, eds. Critical perspectives on user involvement. Bristol: The Policy Press, 2012:33–45.

[61] Peat M, Entwistle V, Hall J, et al Scoping review and approach to appraisal of interventions intended to involve patients in patient safety. J Health Serv Res Policy 2010;15(Suppl 1):17–25. 10.1258/jhsrp.2009.009040 20075123

[62] Greenhalgh T, Humphrey C, Woodard F User involvement in health care. Oxford: Wiley-Blackwell, 2011.

[63] Tritter JQ, McCallum A The snakes and ladders of user involvement: moving beyond Arnstein. Health Policy 2006;76:156–68. 10.1016/j.healthpol.2005.05.008 16006004

[64] The Health Foundation. Leading the way to shared decison making. London: The Health Foundation, 2012.

[65] Coulter A, Collins A Making shared decision-making a reality. No decision about me, without me. London: The King's Fund, 2011.

[66] McLean A Empowerment and the psychiatric consumer/ex-patient movement in the United States: contradictions, crisis and change. Soc Sci Med 1995;40:1053–71. 10.1016/0277-9536(94)00179-W 7597459

[67] Boyle D, Slay J, Stephens L Public services inside Out. Putting co-production into practice. London: NESTA, 2010.

[68] Batalden M, Batalden P, Margolis P, et al Coproduction of healthcare service. BMJ Qual Saf 2016;25:501 17. 10.1136/bmjqs-2015-004315 PMC4941163 26376674

[69] Slay J, Robinson B In this together; Building knowledge about co-production. London: New Economics Foundation, 2011.

[70] Alakeson V, Bunnin A, Miller C Coproduction of health and wellbeing outcomes: the new paradigm for effective health and social care. London: OPM, 2013.

[71] Mulley A, Trimble C, Elwyn G Stop the silent misdiagnosis: patients’ preferences matter. BMJ 2012;345:e6572 10.1136/bmj.e6572 23137819

[72] Social Care Institute for Excellence (SCIE). Co-production: an emerging evidence base for adult social care transformation. London: SCIE, 2009.

[73] Herrin J, Harris KG, Kenward K, et al Patient and family engagement: a survey of US hospital practices. BMJ Qual Saf 2016;25:182–9. 10.1136/bmjqs-2015-004006 PMC4789699 26082560

[74] Health Foundation. Evaluation: what to consider. London: Health Foundation, 2015.

